# Occupational factors and low back pain: a Mendelian randomization study

**DOI:** 10.3389/fpubh.2023.1236331

**Published:** 2023-08-30

**Authors:** Zifeng Wang, Wubing Feng, Qi Jin

**Affiliations:** ^1^Department of Orthopedics, Taicang Shaxi People’s Hospital, Taicang, China; ^2^Department of Orthopedics, Nanjing Medical University Affiliated Wuxi Second Hospital, Wuxi, China; ^3^Department of Orthopedics, No.1 Traditional Chinese Medicine Hospital in Changde, Changde, China

**Keywords:** work, occupational disease, low back pain, Mendelian randomization, instrumental variable, causal inference

## Abstract

**Background:**

Low back pain (LBP) is a common condition and a leading cause of health function loss worldwide. This study assessed the impact of occupational factors on LBP using Mendelian Randomization (MR) method, controlling for confounding variables.

**Methods:**

Based on publicly available genome-wide association studies (GWAS), two-sample univariate and multivariate MR analyses were performed to assess the causal effect of occupational factors on LBP. We used the inverse variance weighted (IVW) method and sensitivity analyses to generate the total results for the univariate MR analysis. Furthermore, we performed multivariate MR analysis to assess the direct causal association between occupational factors and LBP after accounting for potential confounding variables.

**Results:**

The total causal effect of genetically predicted job involves heavy manual or physical work on LBP was found to be significant (IVW OR, 2.117; 95% CI, 1,288–3.479; *p* = 0.003). Upon adjusting for potential confounding variables, the direct effect of job involves heavy manual or physical work on LBP remained statistically significant. Similarly, the total causal effect of genetically predicted job involves mainly walking or standing on LBP was also found to be significant (IVW OR, 1.429; 95% CI, 1,035–1.975; *p* = 0.030). However, upon adjusting for potential confounding variables, the direct effect of job involves mainly walking or standing on LBP became insignificant. In contrast, the findings from the MR analysis indicated a lack of association between work/job satisfaction and LBP. Sensitivity analysis consistently supported these trends.

**Conclusion:**

Our results supported a causal link between job involves heavy manual or physical work and increased risk of LBP, while finding no significant associations between prolonged walking/standing at work, job satisfaction, and LBP, providing valuable insights for the development of targeted prevention and intervention strategies for LBP.

## Introduction

Low back pain (LBP) is a prevalent condition frequently encountered in orthopedic clinics, and its origin is multifactorial. The onset of this condition can be attributed to soft tissue injury or irritation, intervertebral disk damage, vertebral body trauma, articular process injury, and neurovascular structure damage. However, it can be further aggravated by a multitude of factors such as psychosocial issues, obesity, and trauma (1). As per a recent study on the global burden of disease, it has been found that LBP is the primary cause of loss of health function worldwide (2). The global incidence of LBP has been approximated to range from 1.4 to 20.0% (3). Given the significant incidence of LBP and its potential for causing disability, there is a pressing need to identify risk factors associated with this condition. Such identification could have far-reaching implications for public health and contribute to enhancing overall quality of life.

There exists a strong correlation between work and health, with excessive work being associated with an elevated likelihood of developing certain ailments, including depression (4) and cardiovascular disease (5). The issue of LBP that arises from work-related activities is a matter of significant concern. Studies have indicated that this type of pain may be linked to physically demanding work (6, 7), prolonged periods of standing and walking while on the job (8), and job satisfaction (9). Despite the abundance of observational studies examining the correlation between work and LBP, the underlying causal relationship between occupational factors and the onset of LBP remains elusive due to residual confounding and reverse causation.

Mendelian randomization (MR) is a novel statistical approach that employs distinct single nucleotide polymorphisms (SNPs) as instrumental variables (IVs) to establish a credible causal relationship between phenotype (exposure) and disease (outcome). This method has the potential to mitigate the impact of confounding variables and reverse causality, as reported in the literature (10). The multivariate Mendelian randomization approach is a recent development in the field of MR. It involves an expansion of the univariate MR approach, which enables the investigation of the direct causal impact of a variable on an outcome while accounting for the influence of another variable. This approach is useful in mitigating the effects of genetic pleiotropy and in drawing impartial conclusions (11). Given the intricate nature of the etiological factors associated with LBP, which may encompass a multitude of variables such as socioeconomic status, psychological factors, unhealthy lifestyle practices, and medical conditions (12), these factors have the potential to obscure the causal link between occupational activities and LBP. The current study incorporated various factors that have been previously reported in the literature, including obesity (13), smoking (14), pathological psychology (15), physical activity (16), sedentary behavior (17), sleep (18), and education (19), for the purpose of adjustment in the model. These factors have been identified as potential confounders to the outcome of interest and were therefore deemed relevant for inclusion in the analysis.

The present study aimed to evaluate the causal impact of occupational factors on LBP through a two-sample univariate MR approach. Additionally, a multivariate MR approach was employed to identify and control for potential confounding effects, thus ensuring the reliability of the results. The disentanglement of intricate causal connections between occupational variables and LBP will bear significant implications for enhancing the safeguarding of workers from occupational injuries and mitigating health inequalities in society.

## Methods

### Study design

**Figure 1:**
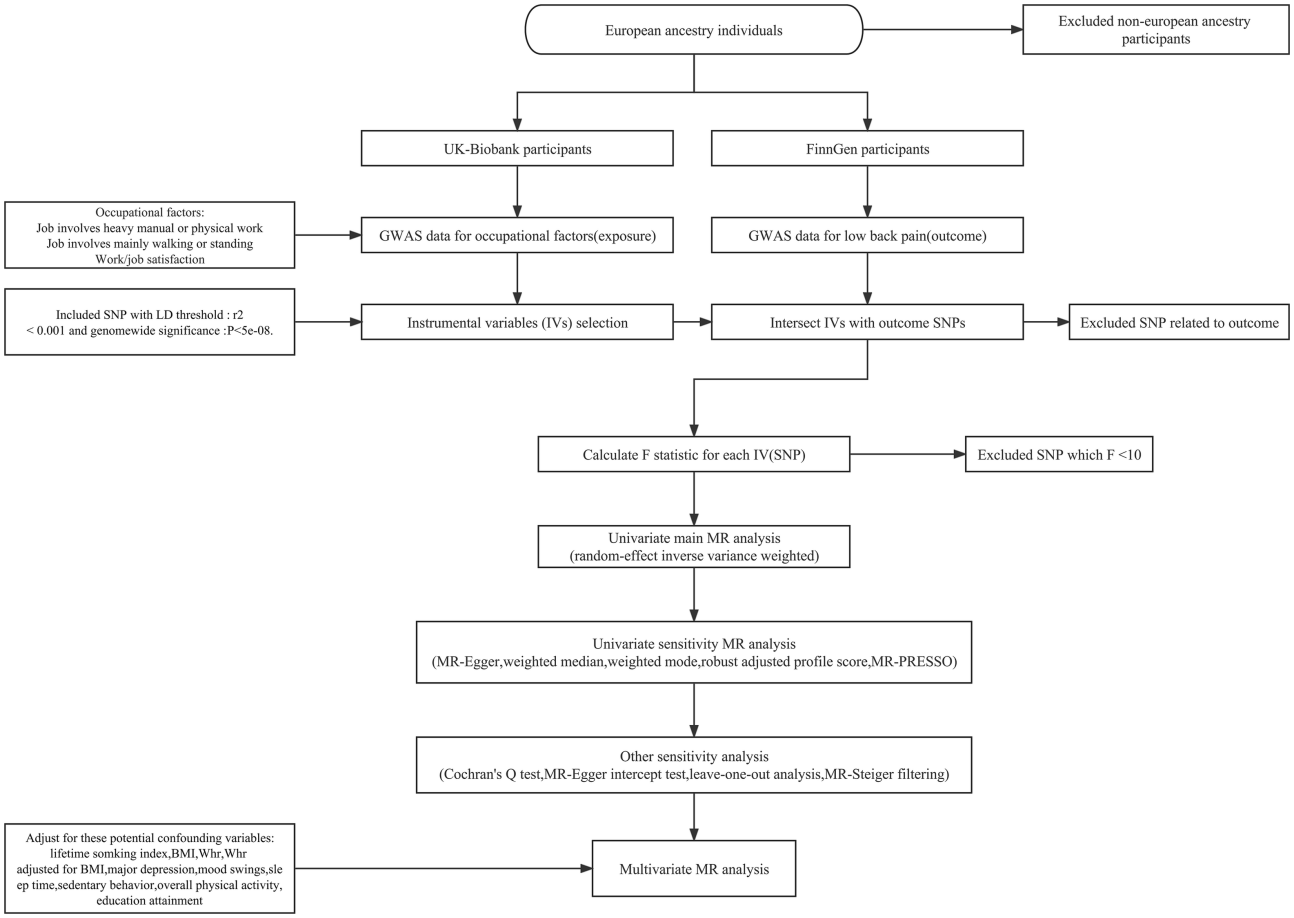
The flowchart of the Mendelian randomization study design.

The present study utilized summary-level data from published genome-wide association studies (GWASs), as well as data from the UK Biobank study and the FinnGen consortium, to conduct a two-sample MR analysis. The cohorts for both exposure and outcome were limited to individuals of European ancestry in order to mitigate the influence of population stratification bias. Initially, we employed univariate MR to estimate the causal effects of the three occupational factors and LBP for the genetic predictions. Subsequently, a multivariate MR framework was employed to evaluate the direct causal impact of the three aforementioned lifestyle factors on the likelihood of experiencing LBP. All studies that were incorporated in the analysis had obtained approval from the respective ethical review boards and the participants had provided their informed consent. Consequently, the present study did not necessitate any supplementary ethical approval. The flowchart of the study design is presented in Figure 1.

### Data sources for exposures

The MRC-IEU GWAS pipeline was utilized to produce genetic statistics at a summary level pertaining to occupational factors. The study sample consisted of 263,615 individuals of European descent from the UK-Biobank cohort. The phenotypic characterization of occupational conditions in this study was derived from self-reported categorical variables that were ordered. The study’s participants were administered a touch screen-based questionnaire that inquired about their occupational involvement in physically demanding or labor-intensive tasks. The specific question posed was, “Does your work entail heavy manual or physical labor?” The inquiry with IEU code ukb-b-2002 pertains to the extent of physical activity involved in one’s work, while the IEU code ukb-b-4461 represents whether the individual predominantly entails walking or standing. The IEU code ukb-b-2105 pertains to an inquiry regarding job satisfaction. The response scale utilized in the study consisted of six options, ranging from 1 to 6, which corresponded to the following categories: “Never/rarely,” “Sometimes,” “Usually,” “Always,” “Do not know,” and “Prefer not to know.” Responses indicating “do not know” or “prefer not to answer” were treated as missing values. The UK Biobank conducted adjustments for age, sex, and a maximum of 20 principal components of ancestry in the association tests. Detailed information for exposures is available in Table 1.

**Table 1:** Information for exposures and outcomes.

Phenotype	Unit	Sample size	Ancestry	Consortium/cohort	Author	Year of publication/release	PubMed ID
Job involves heavy manual or physical work	Categorical ordered	263,615	European	MRC-IEU	Ben Elsworth	2018	–
Job involves mainly walking or standing	Categorical ordered	263,556	European	MRC-IEU	Ben Elsworth	2018	–
Work/job satisfaction	Categorical ordered	105,358	European	MRC-IEU	Ben Elsworth	2018	–
Lifetime smoking index	SD	462,690	European	UK Biobank	Wootton et al.	2019	31689377
BMI	SD	806,834	European	GIANT + UK Biobank	Pulit et al.	2018	–
WHR	SD	697,734	European	GIANT + UK Biobank	Pulit et al.	2018	–
WHR adjusted for BMI	SD	694,649	European	GIANT + UK Biobank	Pulit et al.	2018	–
Major depression	Binary	500,199 (170,756 cases)	European	PGC + UK Biobank	Howard et al.	2019	30718901
Mood swings	Binary	451,619 (204,412 cases)	European	MRC-IEU	Ben Elsworth	2018	–
Sleep time	SD	91,105	European	UK Biobank	Doherty et al.	2018	30531941
Sedentary behavior	SD	91,105	European	UK Biobank	Doherty et al.	2018	30531941
Overall physical activity	SD	91,105	European	UK Biobank	Doherty et al.	2018	30531941
Education attainment	SD	766,345	European	SSGAC + UK Biobank	Lee et al.	2018	30038396
Low back pain	Binary	273,994 (25,163 cases)	European	FinnGen	Kurki et al.	2022	–

BMI, Body Mass Index; WHR, Waist-to-Hip Ratio; MRC-IEU, The MRC Integrative Epidemiology Unit; GIANT, Genetic Investigation of ANthropometric Traits Consortium; PGC, Psychiatric Genomics Consortium; SSGAC, Social Science Genetic Association Consortium.

### Data sources for potential confounders

A total of 10 characteristics were considered as potential sources of confounding. We used the Lifetime Smoking Index (20) to measure the behavioral characteristics of smoking in individuals, which combines smoking indicators with a simulated half-life constant into a formula in which indicators include smoking status (current, past, never), age at smoking initiation, number of cigarettes smoked per day and time to quit. This GWAS included 462,690 participants from the UK-Biobank cohort, and each standard deviation (SD) increase in the lifetime smoking index was equivalent to a person smoking 20 cigarettes per day for 15 years and quitting 17 years ago, or a person smoking 60 cigarettes per day for 13 years and quitting 22 years ago. Obesity phenotypes were derived from the GWAS conducted by Puilt et al. and included body mass index (BMI, body weight [kg]/standing height squared [m^2^]), which describes general obesity, and waist-to-hip ratio (WHR, waist circumference [cm]/hip circumference [cm]) and WHR adjusted for BMI (WHRadjBMI). We used two phenotypes to measure participants’ pathological psychological status, major depression and mood swings. Regarding major depression, we used GWAS summary data from the Psychiatric Genomics Consortium (PGC) for major depression, which comprises 130,664 major depression cases and 330,470 controls of European ancestry by Howard et al. (21). Mood swings are used to define the tendency for frequent, sudden and unpredictable changes in mood states to assess an individual’s personality traits and anxious and stressful psychological states, and the phenotype is based on a questionnaire asking UK Biobank participants, “Do your moods fluctuate frequently?,” to which participants could answer “yes” or “no” (22). The phenotypes of sleep time, sedentary habits and total physical activity in daily life were objectively measured using a wearable accelerometer (23). Finally, educational attainment was obtained from the Social Science Genetic Association Consortium (SSGAC) GWAS meta-analysis of participants’ reported years in school at age 30, with different educational qualifications recalculated according to the International Standard Classification of Education to derive the corresponding years of education.

### Data sources for LBP

To minimize the risk of type I error due to sample overlap between exposure, mediating factors and outcome, we used the genetic tools for LBP from the latest large sample GWAS meta-analysis (round 8) conducted by the FinnGen consortium, which included 273,994 participants (25,163 events and 248,831 controls). The LBP cases were defined as based on codes M54.5 in the International Classification of Diseases, 10th Revision (ICD-10) and 724.2 in ICD-9. The FinnGen study excluded individuals with unclear sex, high genotype deletion rates (>5%), excessive heterozygosity (±4 SD) and non-Finnish ancestry to ensure data quality and homogeneity. In addition to these exclusions, association tests were carefully adjusted for various covariates, including age, sex, 10 genetic principal components of ancestry and genotyping batches. All information on the sources of GWAS data on exposure and outcome can be found in Table 1.

### Instrument identification and Mendelian randomization

Initially, a two-sample univariate MR analysis was performed to investigate the impact of occupational variables, specifically “engagement in physically demanding or manual labor,” “predominantly involving walking or standing,” and “satisfaction with work/employment,” on the comprehensive causal influence of LBP. Furthermore, multivariate Mendelian randomization analyses were performed to evaluate the autonomous causal impact of each exposure on LBP and to account for plausible confounding factors. In order to satisfy the fundamental assumptions of MR analyses, which require that genetic variants utilized as instruments are consistently linked to the exposure of interest, do not display any correlation with confounding factors that influence the relationship between exposure and outcome, and are not autonomously linked to LBP through pathways other than the exposure of interest, we opted to choose genome-wide traits within a 10,000-kb range for each trait, using strict pairwise linkage disequilibrium (LD) thresholds (*r*^2^ < 0.001) for genome-wide significant SNPs (*p* < 5e-08). The utilization of European samples derived from the 1,000 Genomes Project as a reference panel for LD is proposed (24). Furthermore, the F-statistic was calculated for each exposure, whereby an F-statistic exceeding 10 was regarded as indicative of adequate instrument strength (25). The F-statistic was calculated as Beta^2^/SE^2^ (26). To obtain causal estimates, we used inverse variance weighted (IVW) as the primary MR method, which combines estimates of the effect of each SNP on exposure on outcomes into causal estimates using random effects meta-analysis to calculate Wald ratios, with standard errors derived using the delta method (27).

### Sensitivity analysis

We used MR-Egger regression (28), weighted median (29), weighted mode (30), and robust adjusted profile score (RAPS) (31) and the Mendelian Randomization Pleiotropy RESidual Sum and Outlier (MR-PRESSO) (32) method to validate the robustness of IVW results in univariate MR analysis. The MR-Egger regression method is able to assess directional polymorphisms and produce estimates of causal effects when polymorphism effects are also considered. However, it depends on the InSIDE and NOME assumption, which requires that the strength of the association between genetic variation and exposure is not correlated with the degree of bias caused by polymorphism and is sometimes of insufficient statistical power (28). The weighted median method is a statistical tool for estimating the median of a weighted empirical density function of ratio estimates that provides a consistent estimate of the causal effect if at least 50% of the instrumental variables are valid and no single IV contributes more than 50% of the weight (29). The weighted mode method could produce robust estimates of causal effects for horizontal polymorphisms even when most IVs were invalid (30). MR-RAPS is designed to address the problem of systematic and specific multivariate variability, it involves balanced multivariate effects and allows for some large values. This approach is particularly useful when no genetic variants satisfy the exclusionary restriction assumption. MR-RAPS could yield reliable estimates even when instrumental variables are weak (31). MR-PRESSO is a statistical method that detects and corrects for outlier genetic variation in Mendelian randomization studies to mitigate bias in causal effect estimates. The method uses a regression-based approach to identify outliers and provide outlier-corrected estimates of causal effects based on a test for heterogeneity in causal estimates of different genetic variants to reduce the effect of outliers (32). Further sensitivity analyses included the MR-Egger intercept test, the MR-PRESSO global test, the Cochran’s Q test, the leave-one-out analysis, and the MR Steiger filtering to verify the robustness of the MR analysis. The MR-Egger intercept analysis was used to assess whether there was horizontal pleiotropy for multiple instrumental variables, and if the intercept term of the MR-Egger regression was significantly different from zero, it indicated that there was horizontal pleiotropy (28). The MR-PRESSO global test is another method of determining the presence of horizontal pleiotropy, which assesses the degree of horizontal pleiotropy by calculating the sum of the squared residuals of the effect of each SNP and the IVW result after removing that SNP (32). We used Cochran’s Q test to identify the presence of heterogeneity (33), When *p*-values significantly indicated the presence of heterogeneity, MR analysis was carried out using the random effects IVW method. We used leave-one-out analysis to determine whether individual SNPs drive causality, and observe whether there is a significant change in causal effect by sequentially excluding a SNP. To avoid reverse causal effects, we used the MR-Steiger filtering method to test the direction of the potential causal associations (34). All MR analyses were performed using TwoSampleMR package (version 0.5.6) (35), Mendelian Randomization package (version 0.6.0) (36) and MVMR package (version 0.3). MR-PRESSO package (version 1.0). All analyses were based on R software (version 4.2.1; The R Foundation for Statistical Computing, Vienna, Austria).

## Results

**Figure 2:**
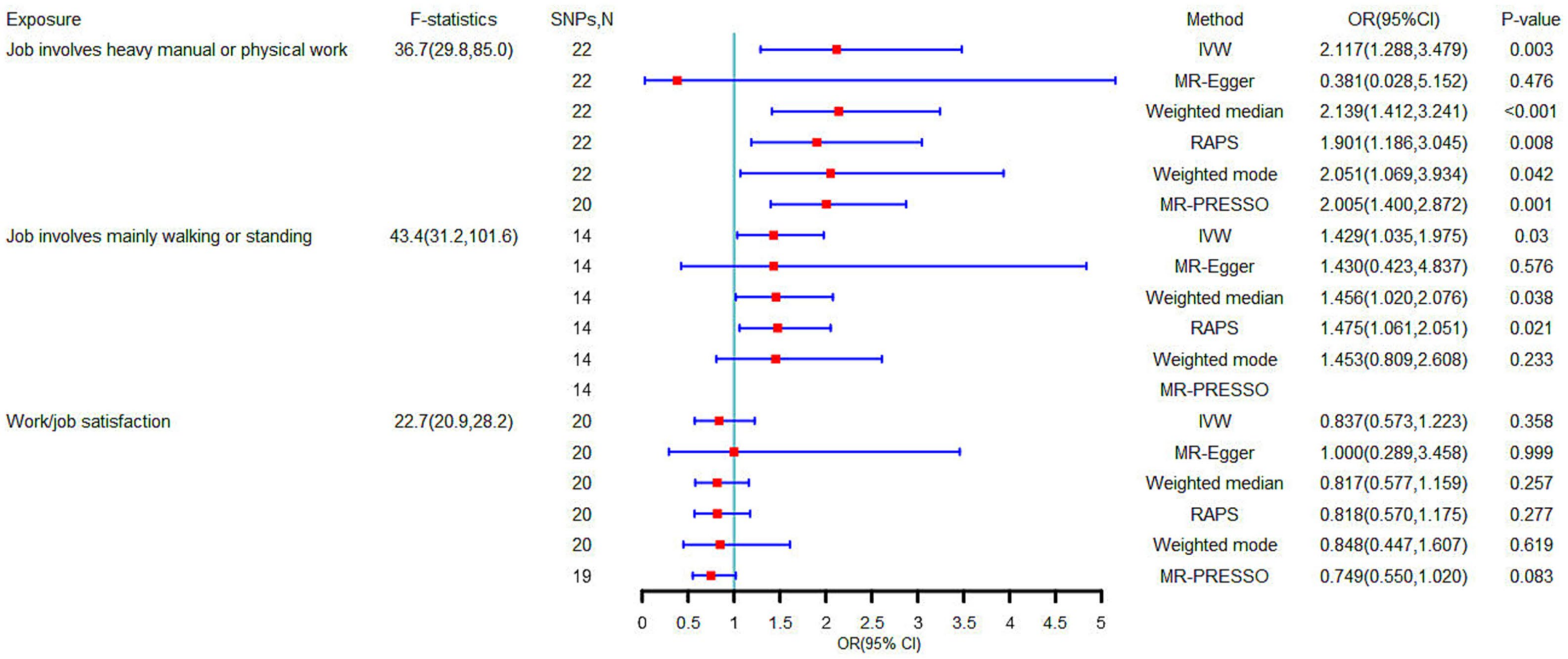
Causal effect of occupational factors on low back pain in univariable Mendelian randomization analyses.

**Figure 3:**
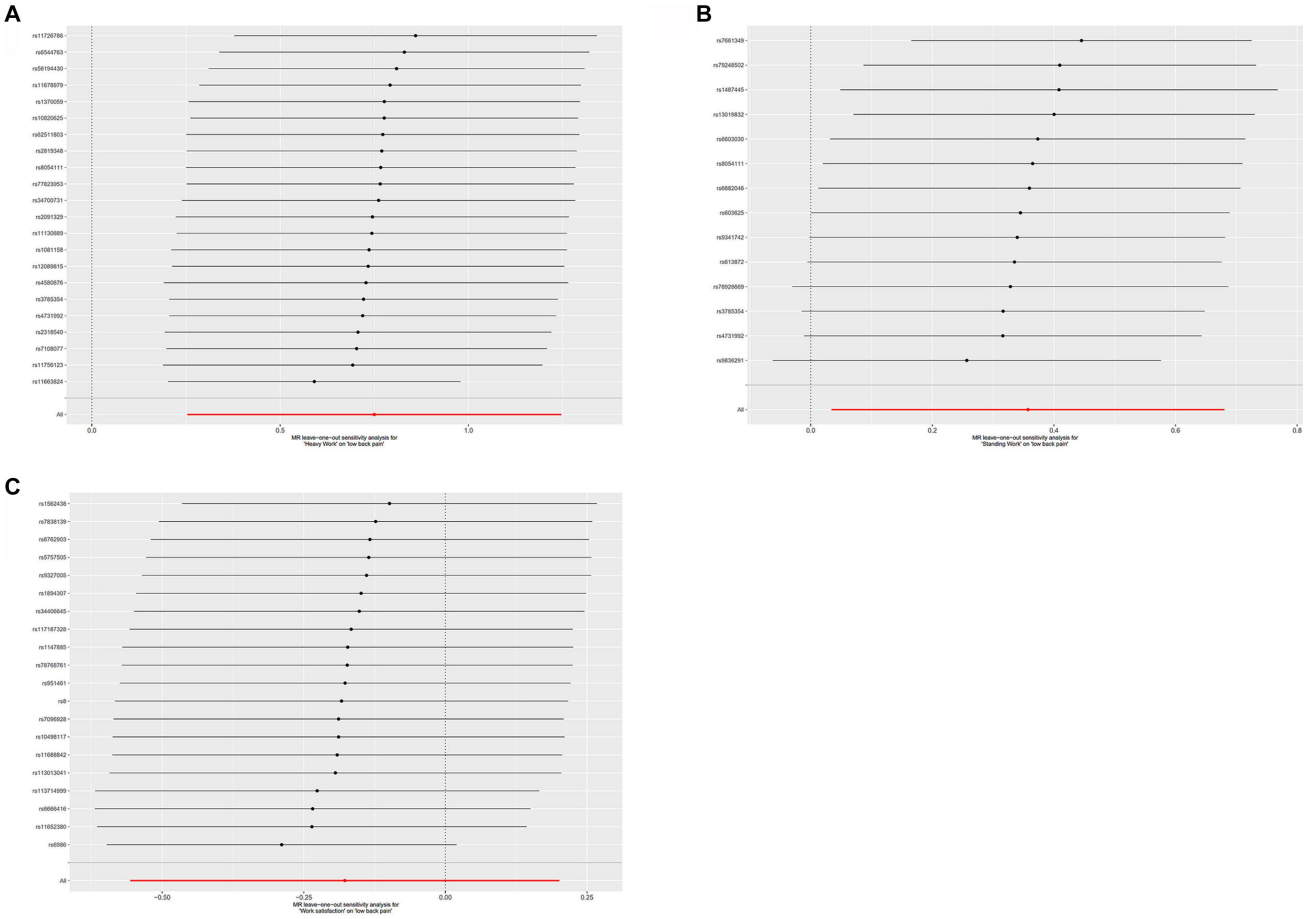
Leave-one-out plots of **(A)** job involves heavy manual or physical work, **(B)** job involves mainly walking or standing, **(C)** work/job satisfaction.

The F-statistics for job involves heavy manual or physical work, job involves mainly walking or standing and work/job satisfaction were 36.7 (from 29.8 to 85.0), 43.4 (from 31.2 to 101.6), and 22.7 (from 20.9 to 28.2), indicating adequate strength of the instrumental variables. Given the significant heterogeneity (*p* for heterogeneity < 0.05) (Table 2), the random effects IVW method was used as the basic analysis. Univariate IVW results showed that genetically predicted jobs involving heavy manual or physical work were significantly positively associated with LBP (OR, 2.117; 95% CI, 1,288–3.479; *p* = 0.003) (Figure 2). Although the MR-Egger intercept test failed to suggest horizontal pleiotropy (*p* for intercept = 0.204), the MR-PRESSO global test showed the presence of horizontal pleiotropy (*p* for global test < 0.001). Robustness analysis using multiple MR methods showed consistent trends, except for MR-Egger, which may be due to violation of the NOME assumption (37). After removing outliers by MR-PRESSO, evidence of horizontal pleiotropy was no longer present (*p* for global test = 0.100), although the results were not significantly different from the original results after removing outliers (*p* for distortion test = 0.702). The leave-one-out analysis showed that all error lines after removing any SNP were located to the right of 0, which demonstrated that causal effects are not driven by partial SNPs (Figure 3A).

**Table 2:** The results for sensitivity analysis.

Exposures	Heterogeneity test		Pleiotropy test				MR-PRESSO distortion test	MR-Steiger directionality test
	Cochran’s Q statistic	*p*	MR-Egger regression		MR-PRESSO global test		*p*	
			Intercepts	*p*	*p* (before removing outliers)	*p* (after removing outliers)		
Job involves heavy manual or physical work	66.99	<0.001	0.029	0.204	<0.001	0.100	0.702	Correct causal direction
Job involves mainly walking or standing	23.41	0.037	−1.12e-05	0.999	0.052	–	–	Correct causal direction
Work/job satisfaction	54.97	<0.001	−0.004	0.771	<0.001	0.017	0.831	Correct causal direction

According to the univariate IVW results, genetic prediction of job involves mainly walking or standing was significantly positively associated with LBP (OR, 1.429; 95% CI, 1,035–1.975; *p* = 0.030) (Figure 2). The MR-Egger intercept test failed to suggest horizontal multiplicity (*p* for intercept = 0.999), and similarly, the MR-PRESSO global test showed the absence of horizontal pleiotropy (*p* for global test = 0.052). Furthermore, although robustness analysis using multiple MR methods showed a consistent trend, leave-one-out analysis showed that this causal effect was driven in part by SNPs (Figure 3B).

When exposure was genetically predicted by work/job satisfaction, neither univariate IVW nor other MR methods were significant, demonstrating a lack of relevant evidence for a causal link (Figures 2, 3C).

Despite the use of the Steiger filtering method, however, for the three exposures of interest, no SNP was found to explain more variance in the outcomes than exposure, i.e., there were no reverse causal IVs. The MR-Steiger directionality test indicated that the hypothesized direction of causality for work-related LBP risk was correct for all (Table 2).

**Figure 4:**
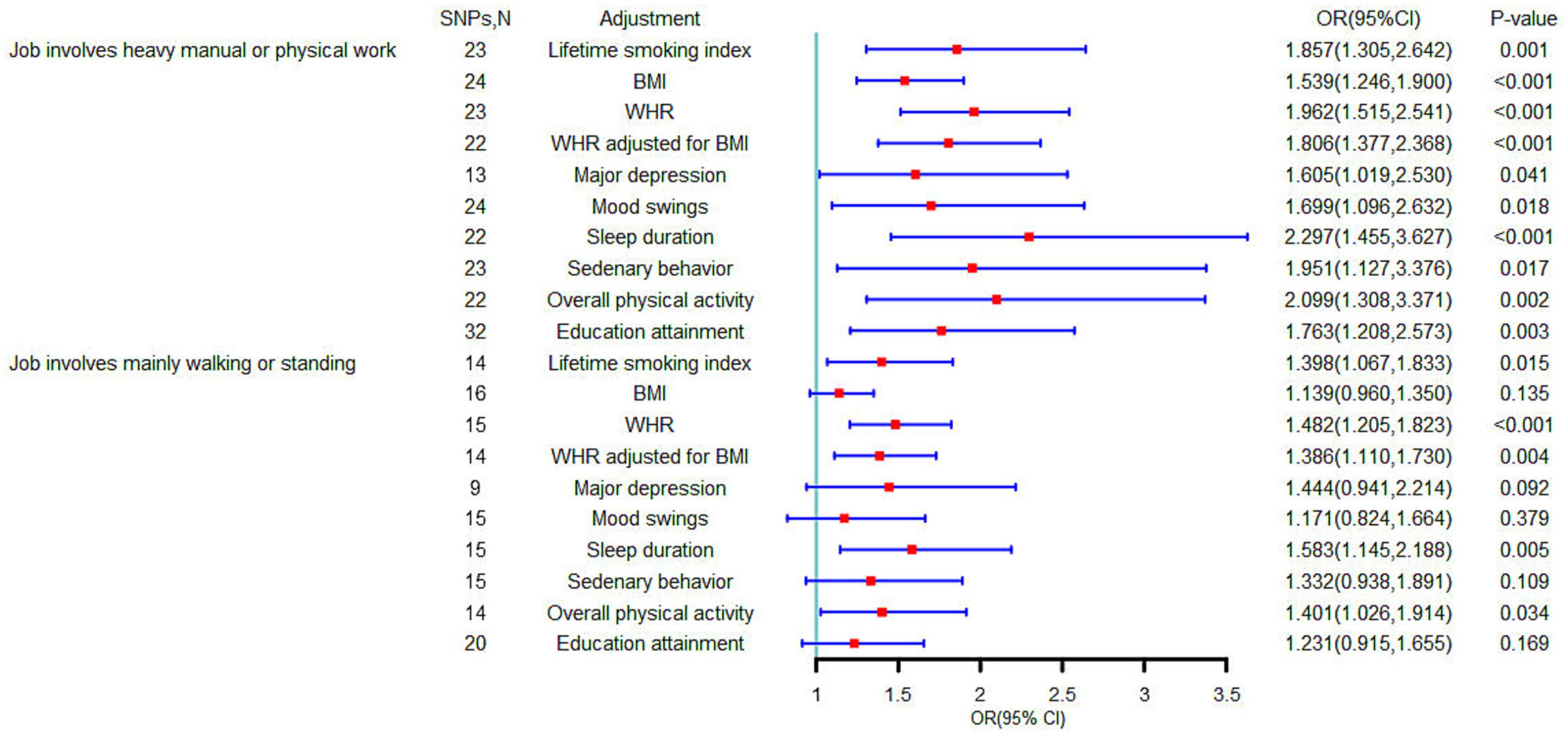
Causal effect of occupational factors on low back pain in multivariable Mendelian randomization analyses (Estimates based on multivariable inverse variance weighted method).

We examined the effect of potential confounders on causal associations using the multivariate MR approach. The direct causal association of job involves heavy manual or physical work on LBP remained significant regardless of adjustment for any of the confounders, although some estimates were attenuated (Figure 4). In addition, when adjusting for BMI, major depression, mood swings, sedentary behavior and years of schooling, the causal relationship between job involves mainly walking or standing and LBP was no longer significant (Figure 4). Given that no causal effects were found for work/job satisfaction and LBP, multivariate MR analyses were not conducted.

## Discussion

This MR study provided evidence that genetically predicted job involves heavy manual or physical work may be causally associated with increased risk of LBP, even after adjusting for various potential confounding factors. By way of contrast, genetically predicted job involves mainly walking or standing and genetically predicted job satisfaction, were not found to be causally linked to the likelihood of experiencing LBP. Our study validated the association between heavy physical work and LBP through a causal inference approach based on genetic data, providing new evidence for occupation-related LBP.

With regard to the positive causal association from jobs involving heavy manual or physical work to LBP, the results are similar to those of a recent national health questionnaire study, which found that occupations such as construction workers and floor cleaners had the highest prevalence of low back pain (frequent and severe), while construction and extractive occupations reported the highest work-related prevalence of LBP, and in addition, workers who required frequent exertion or standing were more likely to report low back pain outcomes than workers who did not report these problems (38). This phenomenon may be attributed to the fact that heavy manual workers have to be exposed to more lifting, dragging, and whole-body vibration, adding additional forces to the spine, and several case-control studies have demonstrated that peak lumbar shear forces during manual labor (39) and cumulative lumbar disk compression are significantly associated with LBP (40), and another cohort study that included 1,131 workers showed that each unit increase in cumulative lower back moment pair would increase the risk of LBP by 3.01-fold after adjusting for age, sex, BMI, smoking, and work status (41). A recent *in vitro* study showed that abnormal spinal mechanical stress activates the Wnt signaling pathway in disk tissue, accelerating disk matrix degeneration, neural invasion, and scorching, ultimately leading to disk degeneration (42).

Our study showed no causal relationship between job involves mainly walking or standing and LBP. Although the IVW results were significant, the leave-one-out method suggested that this causal effect was spurious and that there were several single SNPs driving the causal effect. The multivariate MR approach further explored the effects of potential confounding, and after adjusting for BMI, major depression, mood swings, sedentary behavior and years of schooling, the direct causal effect from job involves mainly walking or standing to LBP was no longer significant, demonstrating that job involves mainly walking or standing was not a direct causal factor for LBP. Previous observational studies have not consistently concluded whether walking or walking at work contributes to LBP, with one cohort study including 187 workers finding a significant negative association between walking and high LBP intensity, and a non-significant result between standing still and high LBP intensity (43), while another study including 698 blue-collar workers reported a significant positive association between prolonged standing during the day and LBP (β = 0.27) (44). Roffey et al. (8) conducted a systematic review that included 18 original studies and concluded, after assessing them using Bradford-Hill causality criteria, that occupational standing or walking was unlikely to be an independent cause of LBP in workers.

As univariate MR did not find a causal effect between job satisfaction and LBP, we did not conduct multivariate MR to explore possible sources of confounding. Earlier studies have suggested a positive association between low job satisfaction and LBP (9, 45), but this association is subject to challenges of confounding and reverse causation. First, low job satisfaction may be associated with excessive job stress. Even in the absence of organic disease, individuals reporting high levels of stress may report more pain, whereas individuals with different personality traits report low stress and no symptoms despite the presence of objective signs of illness (46). This effect may explain the sometimes strong association between work stress and LBP. Alternatively, back injuries at work may contribute to LBP, which may also lead to reduced job satisfaction, creating uncertainty about the correct interpretation of the association between the two.

Our current study has several advantages. We controlled for bias due to population stratification by limiting the study population to individuals of European ancestry. We conducted several important sensitivity analyses to test the assumptions of the MR model. We selected the most significant independent SNPs identified by the most recent and largest GWAS of work-related phenotypes available, so that all SNPs were strongly associated with the exposure of interest, ensuring the “correlation” hypothesis. Several statistical methods were used to reduce the pleiotropic effect and to satisfy the “exclusion restriction” and “exchangeability” assumptions, and possible confounding was analyzed using multivariate MR methods to further validate the robustness of the results and explore possible levels of sources of pleiotropy.

However, the present study must be interpreted in the context of its limitations. First, because genetic analyses are generally weak at detecting non-linear effects and provide qualitative rather than quantitative information, clinical effects may differ from the effect sizes of our genetic analyses. Second, because causal estimates of binary exposure may represent the average causal effect of disease liability (47), a residual possibility of a common risk factor effect for heavy physical labor and LBP may exist. Given these two limitations, the clinical and biological interpretation of causal findings in this study should be interpreted with caution. Third, due to the unavailability of pooled GWAS data on work factors from other ethnic groups, we used only individuals from European populations in this study, and we were unable to further validate the MR results; further studies extending the analysis to other ethnic populations may help to confirm the generalizability of the results. Further MR studies are needed to confirm the finding that heavy work has a causal risk effect on LBP. Finally, our assessment of occupational factors relied on questionnaires, lacking data tools or standards to quantify the physical demands or intensity of a job. Consequently, potential inaccuracies might arise due to cognitive or other subjective factors among the participants, which could lead to attenuated results.

In conclusion, we provided genetic evidence that heavy physical work could elevate the risk of LBP; at the same time, there was no statistically significant relationship between prolonged walking and standing at work, job satisfaction, and LBP. These findings may contribute to the development of effective LBP prevention and intervention strategies. For instance, encouraging regular breaks for heavy manual workers, optimizing the work environment, providing appropriate tools and equipment, alleviating lower back pressure, and implementing routine health check-ups can be undertaken to ameliorate the health inequalities stemming from lower back pain induced by strenuous physical labor.

## Data availability statement

Publicly available datasets were analyzed in this study. This data can be found here: The original contributions presented in the study are included in the article, if more information is needed, the corresponding author can be contacted.

## Author contributions

ZW: conceptualization, methodology, formal analysis, writing, and visualization. ZW and WF: validation, data curation, and visualization. ZW and QJ: supervision and project administration. All authors contributed to the article and approved the submitted version.

## Conflict of interest

The authors declare that the research was conducted in the absence of any commercial or financial relationships that could be construed as a potential conflict of interest.

## Publisher’s note

All claims expressed in this article are solely those of the authors and do not necessarily represent those of their affiliated organizations, or those of the publisher, the editors and the reviewers. Any product that may be evaluated in this article, or claim that may be made by its manufacturer, is not guaranteed or endorsed by the publisher.
